# “Antarctica just has this hero factor…”: Gendered barriers to Australian Antarctic research and remote fieldwork

**DOI:** 10.1371/journal.pone.0209983

**Published:** 2019-01-16

**Authors:** Meredith Nash, Hanne E. F. Nielsen, Justine Shaw, Matt King, Mary-Anne Lea, Narissa Bax

**Affiliations:** 1 School of Social Sciences, University of Tasmania, Hobart, Tasmania, Australia; 2 School of Humanities, University of Tasmania, Hobart, Tasmania, Australia; 3 Institute for Marine and Antarctic Studies, University of Tasmania, Hobart, Tasmania, Australia; 4 ARC Centre for Excellence for Environmental Decisions, School of Biological Sciences, University of Queensland, St Lucia, Queensland, Australia; 5 School of Technology, Environments, and Design, University of Tasmania, Hobart, Tasmania, Australia; University of Alabama, UNITED STATES

## Abstract

Antarctica is often associated with images of masculine figures battling against the blizzard. The pervasiveness of heroic white masculine leadership and exploration in Antarctica and, more broadly, in Science, Technology, Engineering, Mathematics, and Medicine (STEMM) research cultures, has meant women have had lesser access to Antarctic research and fieldwork opportunities, with a marked increase since the 1980s. This article presents findings from an exploratory online survey examining how 95 women experienced research and remote Antarctic fieldwork with the Australian Antarctic Program. Although women are entering polar science in greater numbers, a key theme of the qualitative findings of this survey is that gendered barriers to participation in research and fieldwork persist. We discuss five key gendered barriers including: 1) Physical barriers, 2) Caring responsibilities/unpaid work, 3) Cultural sexism/gender bias, 4) Lack of opportunities/recognition, and 5) Unwanted male attention/sexual harassment. We argue that the lack of attention paid to gender and sexuality in polar fieldwork contributes to the invisibility and exclusion of women and other marginalized identities broadly. To conclude, we point to the importance of targeted inclusivity, diversity and equity initiatives through Antarctic research globally and specifically by National Antarctic Programs.

## Introduction

Antarctica is a unique setting to contemplate women, leadership, and STEMM, as it has a compelling gendered history [1, 2]. Historically, Antarctica has been a place for the activities and science of white European and North American men–it is a site for heroic masculinity and leadership by men [3, 4]. Women were excluded from exploratory and scientific expeditions, and in the first half of the twentieth century they mainly travelled “South” as wives and partners [5]. Today, Antarctica is supposed to be a workplace for those of all genders. Nevertheless, gendered barriers to participation in research and fieldwork persist, especially for women. This paper presents the qualitative findings of a survey of women’s experiences in Antarctica. Specifically, it identifies five key barriers that persist and contextualizes these within the wider context of fieldwork and research. Throughout this paper, we employ commonly used terms to refer to gender such as “men”, “women”, “male” and “female”. However, we acknowledge that gender is not binary, is socially produced, self-identified, and complex.

### The STEMM pipeline

Women are entering STEMM professions in greater numbers; however, women continue to be underrepresented in senior leadership positions [6]. Women’s underrepresentation in leadership is often attributed to a shortage of women in the STEMM pipeline–a visual metaphor used to describe how individuals become professional scientists/engineers. The pipeline portrays individuals as water flowing through a series of narrowing pipes in which the flow or supply of girls/women shrinks at each junction, representing career stages [7]. At the end of the pipeline, water drips into a cup portraying the small number of women becoming STEMM professionals compared to the number who enter the pipeline. The leaky pipeline describes the loss of women from each transitional stage in a professional science career [8] and is particularly acute in academia. Recent Australian data show that women make up half of STEMM postgraduate students yet represent only 20% of senior STEMM academics [6].

The pipeline metaphor provides scholars with directives to investigate where leaks in the pipeline occur and why. However, the metaphor does not provide a nuanced view of intersectional disadvantage, for example, and the solution to the problem is to “merely patch the leaks” [9]. Whilst most research examining women’s underrepresentation in STEMM has been conducted in the US, there is now an emerging body of interdisciplinary Australian research (e.g. [7, 10]). This research identifies persistent barriers to advancement for women in STEMM in Australia. These barriers include gender bias in hiring and promotion, difficulty accessing networks, masculine management styles, lack of role models and mentors, and lack of support for promotion/advancement. Moreover, women also identify having to negotiate a macho workplace culture characterized by sexual harassment, bullying and sexism, insufficient parental leave policies and flexible work arrangements, and feelings of isolation and invisibility in the workplace. This literature is valuable in providing an Australian context for the status of women in STEMM by identifying structural gender inequalities and persistent barriers to women’s advancement, particularly in fields dominated by men.

### Women in polar science

Historically, modern industrial societies have been organized around the domestic orientation of women due to their association with motherhood and perceived lack of fitness for participation in the public sphere compared to men [11]. In line with this ideology, women have historically been subordinated to men in the context of gender hierarchies in polar research and remote fieldwork [3]. Indeed, many polar institutes have justified the exclusion of women by arguing that there were no facilities for them on stations [3]. It was not until Soviet geologist Maria Klenova began her Antarctic fieldwork in 1956 that things started to change [12]. The first all-women scientific team to work in Antarctica went South in 1969. The significance of this was noted by Walter Sullivan in the *New York Times*, when he described the 1969 expedition of US scientists as “an incursion of females” into “the largest male sanctuary remaining on this planet” [13]. The Australian Antarctic Division and the British Antarctic Survey allowed women to stay on research stations and conduct land-based Antarctic fieldwork starting in the 1980s [14]. Pregnant women and children were welcomed at Argentina’s Esperanza Base starting in 1977 and at Chile’s Villa las Estrellas Base in 1984 as part of each government’s push for territorial claims [15]; in these instances, women’s bodies were manifestations of geopolitical power.

Women’s sexuality was a threatening addition to the homosocial environment populated by male “heroes” of the early days [16]. A homosocial environment is characterized by men’s preference for relations with other men. The physical presence of women also threatened the culture of objectification that had reigned in the early decades of permanent human presence in Antarctica. Collis [14] details how the “Sistine ceiling” of the Weddell Hut at Australia’s Mawson Station–a collage of ninety-two 1970s and 1980s pornographic pinups–was described as a “shrine to the red blooded pioneering spirit” of the earlier explorers and labeled “part of the national heritage” [17]. Wheeler’s Antarctic travel memoir from the early 1990s describes how men used pornography in order to “get a rise” from women on the British Antarctic Survey (BAS) training program at Rothera station [18].

There are two waves of critical literature on women’s presence in Antarctica. Although Land [19] and Chipman [5] published canonical books in the 1980s, the issue of Antarctica lagged behind other feminist issues and was not picked up widely until the following decade. A 1993 Australian conference entitled *Living in Antarctica*: *Women in A Man’s World*? published conference proceedings titled, *Gender on Ice*, and it is here that issues of gender and Antarctica are first explored in depth in the Australian context. The conference was “intended to be provocative” and stimulate discussion about gender in Antarctica, but “the verdict of this conference was that the construct is no longer relevant” [20]. The Spring 2009 edition of *Signs* included another wave of papers, following on from the 2008 conference *Comparative Perspectives Symposium*: *Gender and Polar Studies*. The clustering of articles around conferences and frequent cross-referencing suggests that the implications of women’s presence in Antarctica has not been widely discussed outside of the two meetings. Indeed, in 2009, Lewander [21] observed that “gender research in polar history with regard to Antarctica is still comparatively rare”. Nevertheless, the scholarly landscape is changing as women become more visible in Antarctica and more recently are integrated into the everyday activities of National Antarctic Programs. Today nearly 60% of early career polar researchers are women [12]. Although an emerging body of scholarly work provides a renewed focus on the gendered context of polar research and fieldwork (e.g. [3]), as we discuss in this article, gender bias remains in Antarctic science and fieldwork.

### Antarctic fieldwork for women

Observing and quantifying phenomena in the field is core to many science disciplines. Yet, fieldwork is an activity that problematically highlights a discipline’s masculinist underpinnings [22]. Namely, the ideal scientific fieldworker is discursively produced as a white, able-bodied, fit man who “conquers” the (feminine) terrain [23]. In relation to the feminization of Antarctica, Collis [14] attests:

The Australian Antarctic Territory was, and remains, a space onto which fantasies of an idealized Australian masculinity have been projected: the final frontier awaiting penetration…

The highly gendered character of fieldwork means that it can be a risky activity for women in any scientific field [24]. For instance, recent research shows that women in STEMM are 3.5 times more likely to experience harassment in the field compared to men [25]. Emerging STEMM gender equity initiatives coupled with the recent #metoo hashtag on social media have arguably provided new platforms for women in STEMM to report sexual harassment and to more openly challenge problematic science research cultures [26].

There is now an emerging body of research focusing on women’s experiences of remote scientific fieldwork [25]. However, there is relatively little research focusing on gender equity and diversity in Antarctic research and fieldwork [3, 27]. With a few exceptions, much of this work draws upon women’s historical rather than contemporary experiences. To address this gap, using Australian Antarctic research as a case study, this article builds on the existing remote fieldwork literature to inform long-term responses to gendered inequality in the field. It specifically focuses on women’s experiences within the Australian Antarctic Program. Australian Antarctic research activities, including those undertaken by researchers from universities and government agencies, is primarily supported logistically and financially by the Australian Antarctic Program and administered through the Australian Antarctic Division (AAD). The Program operates research stations, ships, aircraft, and field support capabilities, including the provision of workplace health and safety training and employment of station staff.

## Methods

This study used an online survey and a qualitative approach to address the following key research questions:

1. What are the socio-demographic characteristics of women working in Australian Antarctic research?
2. What are the research/fieldwork experiences of women working in Australian Antarctic research?

This study is exploratory and flags areas where future research, including further quantitative analysis, is necessary.

## Recruitment

Inclusion criteria were: Women (aged 18+) working presently or in the past in Australian Antarctic research who had been to Antarctica at least once with the Australian Antarctic Program (from the 1950s to present) either on a research vessel to the Southern Ocean or to a research base or field camp. Australia has three permanent continental bases–Mawson, Casey, and Davis–as well as the sub-Antarctic Macquarie Island base. The stations host logistics hubs and living quarters. The experiences of both those who spent a summer season (typically November-March) and those who wintered-over were considered. Over-winter populations at each base are typically 10–30 people, with numbers increasing to up to 100 over summer. Summer fieldwork can take place in a remote camp away from the main base. While these work environments and the length of time spent in Antarctica differ depending on location, all were included to consider the experiences of the widest possible range of women. Antarctica was defined to include the Antarctic continent, sub-Antarctic Islands, and the Southern Ocean.

To our knowledge, there are no publicly available data detailing the total number of women who have participated in Australian National Antarctic Research Expedition (ANARE) and AAD research and fieldwork. Between 1858 and 1984, 86 Australian women travelled to Antarctica to accompany their husbands or to work in various capacities, some travelling to the continent multiple times [5]. The first women overwintered in the Antarctic as part of the Australian program in 1981.Women now comprise approximately 25% of the over-summer personnel and in the austral summer field season 2017/18, about 40% of the scientists in the Australian Antarctic program were women [28].

Participants were recruited mainly through direct email to members of relevant Antarctic networks and associations (e.g. the Association of Polar Early Career Scientists (APECS), Women in Polar Science). The email distributed to relevant groups contained a link to the survey and an open invitation to participate. We also recruited participants via social media feeds created specifically for this study. Participants were directed via web link to an information sheet that provided detail on the background, rationale, and anticipated outcomes of the project. Participants were self-selected and are not necessarily representative of the entire population of women who have worked in Antarctica with the Australian Antarctic Program.

The exploratory voluntary survey contained 57 questions and was hosted on the Survey Monkey platform (San Mateo, CA, USA) (see S1 File). The survey was distributed in October 2017 and was open for one month. To ensure participants could provide informed consent prior to participation, an electronic consent form was positioned at the start of the survey. A skip logic was used to ensure that any participant who did not provide informed consent could not complete the survey. This study was approved by the Tasmania Social Sciences Human Research Ethics Committee, Ethics Ref No: H0016840.

We received 166 survey responses. Analyses are restricted to those participants who provided complete survey data by responding to at least 75% of survey items (n = 95; 57%). The survey consisted of closed and open-ended questions (S1 File). This format allowed participants to provide unrestricted comments rather than selecting from only pre-determined choices. Closed questions were used to gather socio-demographic data (e.g. gender, age, nationality, marital status, ethnic group, income, occupation, and education) and information about relevant gendered experiences of Antarctic fieldwork. Open-ended questions were used to gather data on participants’ perceptions about being a woman in Antarctic research, including experiences of conducting remote field work and sexual harassment. All questions were designed from the relevant literature (e.g. [1, 2, 3, 5, 23, 25]) The questions on harassment are specifically drawn from Clancy et al.’s 2014 study on harassment in academic field experiences [25]. Participants could decline to answer any question. Following Clancy et al. [25], we did not ask participants about specific field sites due to the risk of identifying participants.

A demographic overview of the sample is provided in Table 1. These demographics reflect participant identities at the time of completing the survey. Participants were highly educated (57% with a PhD, n = 55); predominantly in the 30–50 age range (average age = 45) at the time of the survey; and working in a range of scientific, government, and private roles. White middle class, heterosexual women are overrepresented in this sample. Women and men of color and other marginalized identities are especially underrepresented relative to white heterosexual men in polar research [29]. Most women in this sample (63%, n = 60) did not have children and began working in Australian Antarctic research in the last two decades. This is unsurprising given that women were not permitted to undertake fieldwork in Antarctica with the Australian Antarctic Program until the 1980s.

**Table 1:** Participant demographics at time of survey completion.

*Category*	*Number of People*
**Age**	
20–29 years	10
30–39 years	40
40–49 years	24
50–59 years	12
60–69 years	4
**Highest educational level obtained**	
Bachelor’s Degree without Honors	6
Bachelor’s Degree with Honors	11
Graduate Certificate	4
Masters by coursework	6
Masters by research	12
Doctorate by coursework and research	1
Doctorate of Philosophy	55
**Decade began working in Antarctic research**	
1980s	4
1990s	19
2000s	35
2010s	36
**Relationship status**	
Married/in a relationship	68
Single	27
**Number of children**	
0	60
1	13
2	16
3	3
4	2
**Racial/ethnic background**	
White	90
White/Latina	1
South East Asian	1
**Sexuality**	
Heterosexual	72
Queer	1
Bisexual	3
Prefer not to answer	8
**Employment sector**	
Government	44
Private	11
University	35
Other (e.g. self-employed)	5
**Employment status**	
Postgraduate student	13
Technician or field assistant	9
Postdoctoral fellow	12
Government scientist	14
Professor	6
Other (e.g. medical doctor, retired)	40

### Analysis

Qualitative analysis involved developing relevant themes that reflected the qualitative data. We also drew on participants’ demographic data and responses to closed questions to identify associations between separate themes [30]. Loosely informed by a Grounded Theory approach [31], data were analysed by one researcher (MN) using open coding, taking note of any striking words, phrases, and themes in the data. Once common themes were identified, thematic categories were created and relevant data were coded to those categories. Thematic analysis identified gendered barriers to participation in fieldwork as a primary theme in women’s accounts with five sub-themes (e.g. physical barriers) described in detail in the next section. To ensure the validity of this thematic analysis, the codes were discussed and reviewed by the entire research team. The qualitative data are not necessarily representative of all women in the sample, as the comments reflect what women individually chose to write. Nevertheless, simple counts and percentages are used to contextualize the comments and to illustrate the proportion of comments that addressed particular themes. When an issue was raised frequently, weight is attributed to this to reflect an important element of experience. Direct quotes from participants are used to convey the important themes.

## Results

### Barriers to participation in Antarctic fieldwork

A key theme of the data in the survey is gendered barriers to participation in fieldwork. Qualitative findings reveal five key gendered barriers that highlight inequity within both Antarctic research and fieldwork including: 1) Physical barriers, 2) Caring responsibilities/unpaid work, 3) Cultural sexism/gender bias, 4) Lack of opportunities/recognition, and 5) Unwanted male attention/sexual harassment. In the forthcoming sections, our discussion of participant responses reflects the processes, procedures, and culture at the time of their engagement with Antarctic research and fieldwork; and these may have changed over time.

#### Physical barriers

In their open-ended comments, women identified a masculinist “body culture” as a barrier to doing Antarctic fieldwork [23]. One woman noted feeling ill-equipped for the demanding physical tasks required in their role (e.g. work that involved carrying huge and heavy instruments (“I am a small lady”). However, physical capacity was not the main obstacle–seven women note that it is often men’s attitudes about women’s bodily capacities that is limiting (e.g. “potential doubt about physical strength and stamina”; “assumptions are made about how physically fit and capable women expeditioners might be to assist in fieldwork…”; “women seen as poor little girls that needed help with anything they saw as a task for men”). These extracts show that physical strength is an important source of power in the field because it can determine how scientists are selected to work in teams and what tasks they can do. However, this can put women in a bind–they are made to feel like they are not contributing if they are unable to lift a heavy piece of equipment. At the same time, male leaders and supervisors can be reluctant to let women do strenuous tasks out of chivalry. These examples refer to a class of behaviors referred to as “benevolent sexism” in which men “maintain a positive self-image as protectors and providers…”. [11].

I was regularly criticized for being "too independent" when carrying and organizing my own working equipment, yet when I asked for help with heavy loads I was accused of being an "ice princess"…

As Rosner [32] observes, “…supposed feminine inferiority serves as homosocial glue”. This positioning of women as inferior has important career consequences for women because if they are routinely prohibited from doing certain field tasks, their knowledge of certain technologies or machinery may be more limited compared to men. This could affect women’s perceived value as scientists in the future. One respondent reported:

I can’t do fieldwork as part of my project due to the fishing vessel we collect our samples from being made up of 100% men [from another country] and the company that runs the vessel not being comfortable putting me on their ship as a woman, despite me telling them I’m comfortable with it.

As this example suggests, women’s participation in fieldwork may be subject to other cultural assumptions and expectations. Given the international nature of Antarctic research and the difficulty associated with accessing the continent, it is not only Australian infrastructure that impacts upon women’s ability to undertake their projects. National programs often cooperate to share resources or–as in the case of these samples from a fishing vessel–enter arrangements with private operators and receive funding from international philanthropists.

Furthermore, adequate clothing and bodily hygiene are central to survival in Antarctica’s harsh physical conditions. In this study, some women noted that they are often made to feel like physical spectacles in male-dominated fieldwork environments because as women, they stand out as unique or rare but they are also invisible because their bodily needs are frequently ignored [33]. For example, clothing is often not tailored specifically for women’s bodies (e.g. “too big”; men’s sizing only), which can make working difficult and compromise field safety:

Often women are issued with ill-fitting clothing which exposes them to risk (e.g. tight down reduces warming, long sleeves can get caught). Correct fitting has never been taken seriously and it is a discrimination issue that is never engaged with although I have raised it, as others have who are long term and experienced field workers.

Three women pointed out that being in the field can involve “more difficult logistics for sanitary needs” and that basic human activities like urinating “take more consideration” in clothing designed for men. One participant notes that women’s field hygiene historically was a source of “embarrassment” (e.g. “female urinary devices are issued (by the AAD) through a series of whispers and emails rather than with our survival packs”). Personal hygiene during menstruation can be a challenge for women in remote fieldwork because the logistical requirements for washing are not acknowledged in fuel rations (e.g. for heating water). The ability to easily go to the toilet in field clothing or in privacy can be of great consequence to women in male-dominated environments because it can threaten women’s safety and add unnecessary stress to their already demanding jobs [24].

#### Caring responsibilities /unpaid work

Twenty-seven participants (28%) indicated family commitments and caring responsibilities as important sources of inequality in Antarctic research and fieldwork. Thirty-seven percent of participants dedicated 20+ hours to caring responsibilities (e.g. children, elderly parents, animals) each week. According to the most recent Australian time use data, in 2006 women spent approximately the same amount of time on household work (including caring for children, domestic activities, and shopping) as they had in 1992 [34]. In other words, women spend an average of 5 hours per day or 33 hours per week on this work [34]. Although more Australian women have entered the paid labor market in recent decades, their unpaid domestic labor has not declined.

Data collection is at the core of many scientific disciplines and therefore, the practice of a discipline in the field is intimately tied to a scientist’s identity and credibility. Polar scientists are expected to spend weeks or months doing fieldwork on a vessel or research station, often in remote locations [29]. Although women in STEMM often describe fieldwork as a pleasurable aspect of a scientific career [23], participants in this study observe that extended time away from home puts pressure on relationships and makes it difficult to undertake caring responsibilities (especially for sole caregivers) or to plan pregnancies.

…More criticism/comment of a woman’s choice to raise a large (>2 children) family. The same criticism is not directed at men with larger (>2 children) families.

As above, open-ended comments recognize these difficulties occur for men and women but that women bear disproportionate cultural responsibility for caring compared to men [35].

Consequently, women in Antarctic research may struggle to manage their family commitments and meet career expectations. Some women manage by outsourcing the labor of fieldwork to maintain an active research agenda:

My fixed term contract finished. I then had a child and could not re-apply for my previous position as it involved long field work stints in the Antarctic…Having a child effectively halted my Antarctic career as I was no longer able to conduct fieldwork…This was devastating for me as I was, and still am, very passionate about Antarctic research and the project that I was working on…I feel that I am years behind where I was when I couldn’t apply for my (scientific research) position.

Immediately after having my children, I realized I would not be able to do fieldwork in mainland Antarctica due to the duration of the voyage/expedition. There was no other reason. I delegated fieldwork to postdocs/collaborators/students.

However, choosing not to do fieldwork or undertaking local/less time-intensive fieldwork can position women as less committed, entailing additional work to overcome this perception. Such attitudes minimize the contributions made by researchers who are either unable to (due to caring responsibilities or disability) or choose not to travel south (as a growing number are choosing to do because of climate change [36]). As in the extract below, visiting Antarctica continues to confer “a sense of legitimacy which the mere act of going there does not necessarily deserve” [37].

My work has been minimized as not being “real” work experience in Antarctica because I am based indoors (had this by bosses and colleagues).

In addition to caring responsibilities, 44% of participants dedicated 0–5 unpaid work-related hours (e.g. volunteering/work experience, grant writing, publications, emails, mentoring, professional societies, public outreach) to their careers each week. Grant writing and publications are important because they directly impact career trajectories in academia. Similarly, given the difficulty in securing polar fieldwork, women also volunteer or do unpaid work to gain experience. However, the most common unpaid activity for scientists and non-scientists is public outreach. In Australia and elsewhere, it is now increasingly recognized that public outreach–engaging with a lay audience about key scientific issues (e.g. public lectures, school visits, media interviews)–is a critical component of a science career [38]. Most Australian government funding bodies, including the Australian Antarctic Science Program, require that scientists commit to translating their research to the community and there are specific funding schemes dedicated to public engagement. Even though these activities are becoming more valued, the career benefits of public outreach remain unclear [39].

Whilst it is difficult to make a definitive statement about the gendered dimensions of public outreach in Australian Antarctic science from this survey alone, it is useful to consider this issue when examined alongside the number of hours per week that women dedicate to caring responsibilities. It is already well-known that the disproportionate time that women dedicate to caring responsibilities compared to men can significantly impact research productivity [40]. However, a recent survey of academic physicists and biologists reveals that women with children (81%) still do more public outreach work compared to men (50%) with children and men (37%) and women (66%) without children [41]. Dedicating a disproportionate number of unpaid hours to public outreach activities can lead to inequality over the longer term, especially in academia, with outputs such as papers and citations valued more highly.

#### Cultural sexism/gender bias

**Fig 1:**
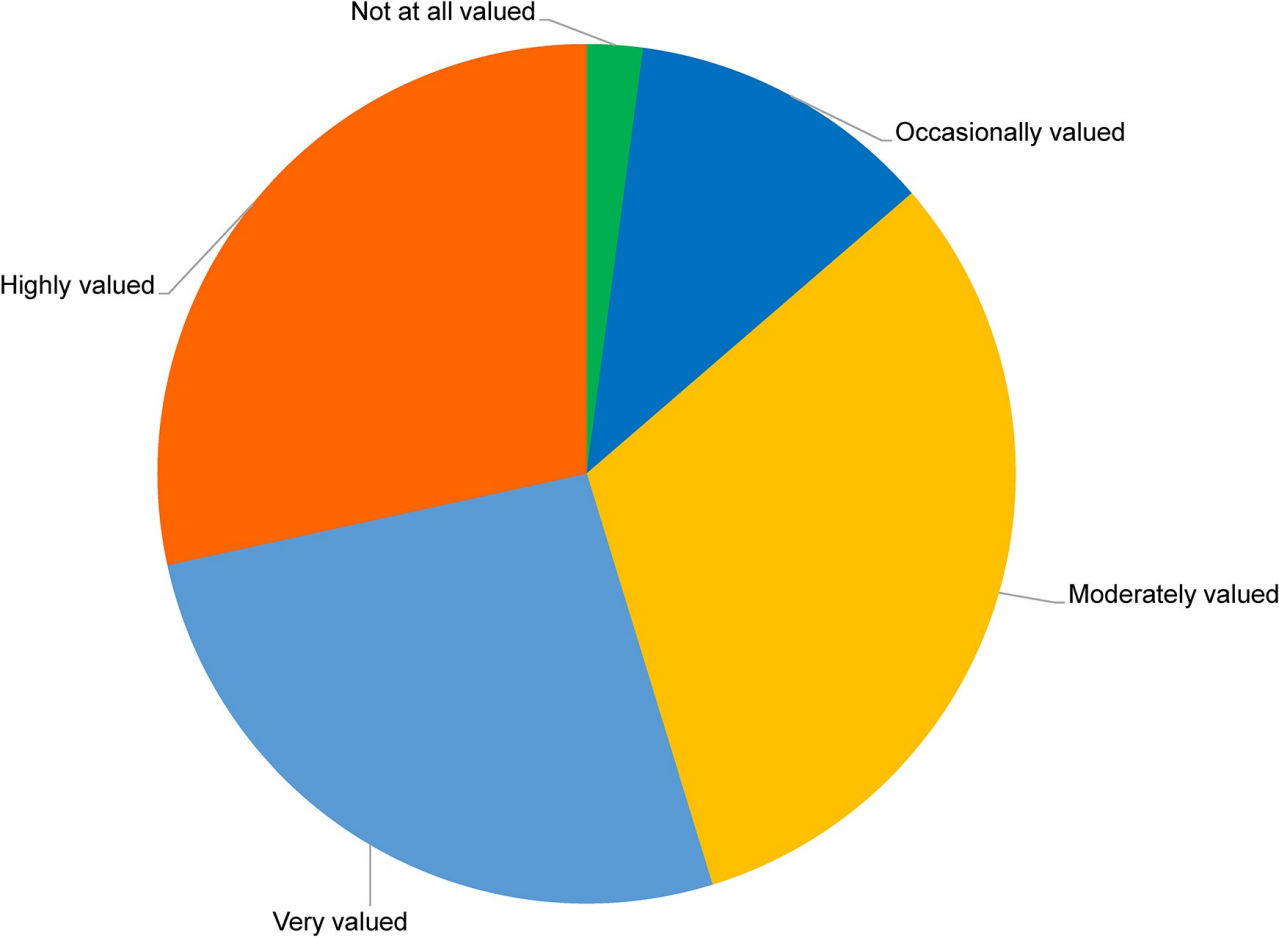
Participant responses to the question “Whilst working within an Antarctic field team how valued was your input regarding the approach, methodology and implementation in your most recent field experience?”

Fifty-two percent of survey participants reported that they were held to the same standard as men (e.g. in terms of research capability, competence in the outdoors, responsibilities, etc.) in their most recent field experience. Twenty-six percent of women reported that they were held to a slightly lower standard compared to me (Fig 1).

**Fig 2:**
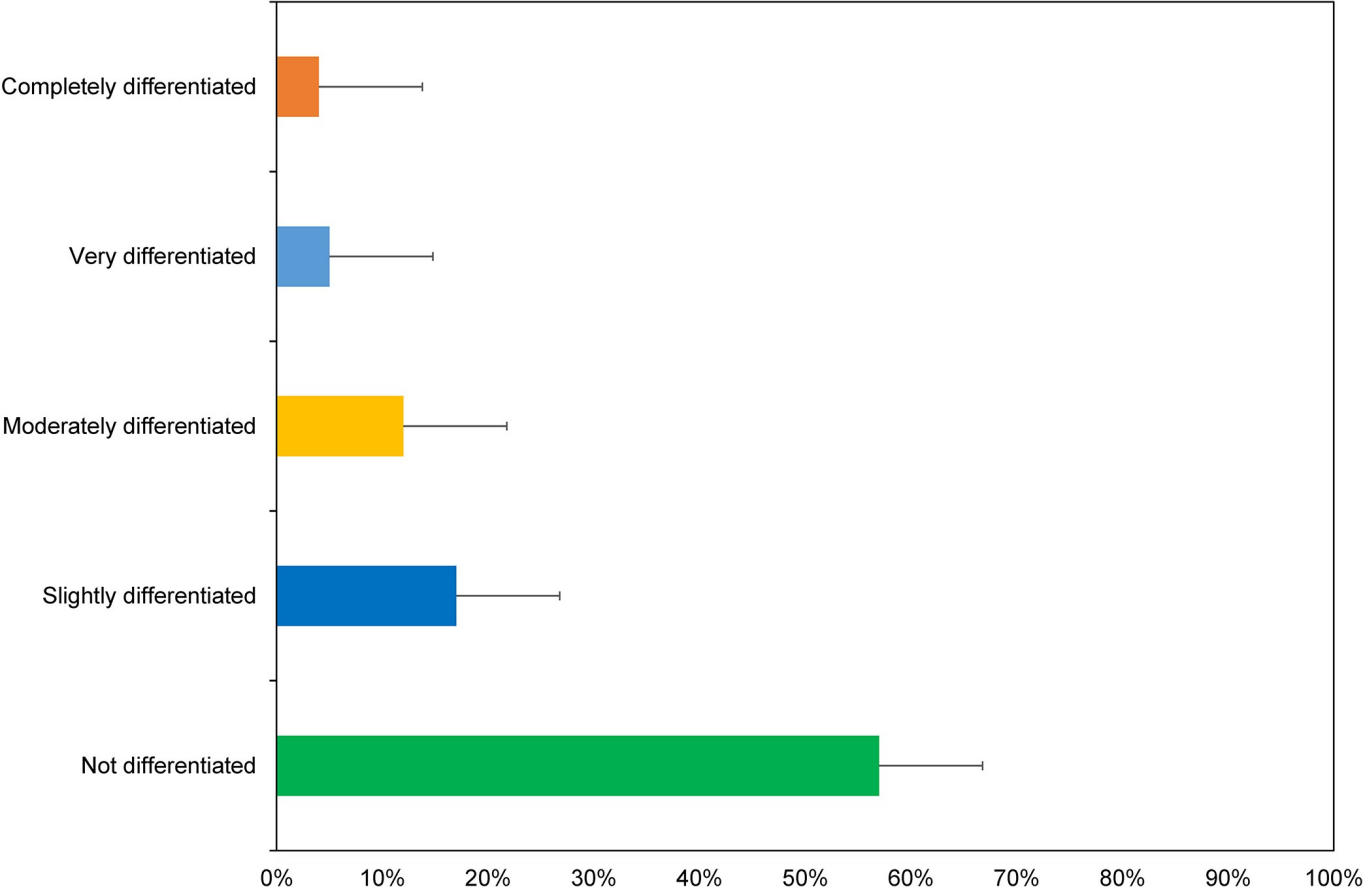
Overall experiences of gender differentiation.

Additionally, 60% of participants report that, in general, their most recent field experience was not gender differentiated (e.g. women and men do separate leisure activities or bureaucratic/cleaning tasks are allocated differently) (Fig 2).

**Fig 3:**
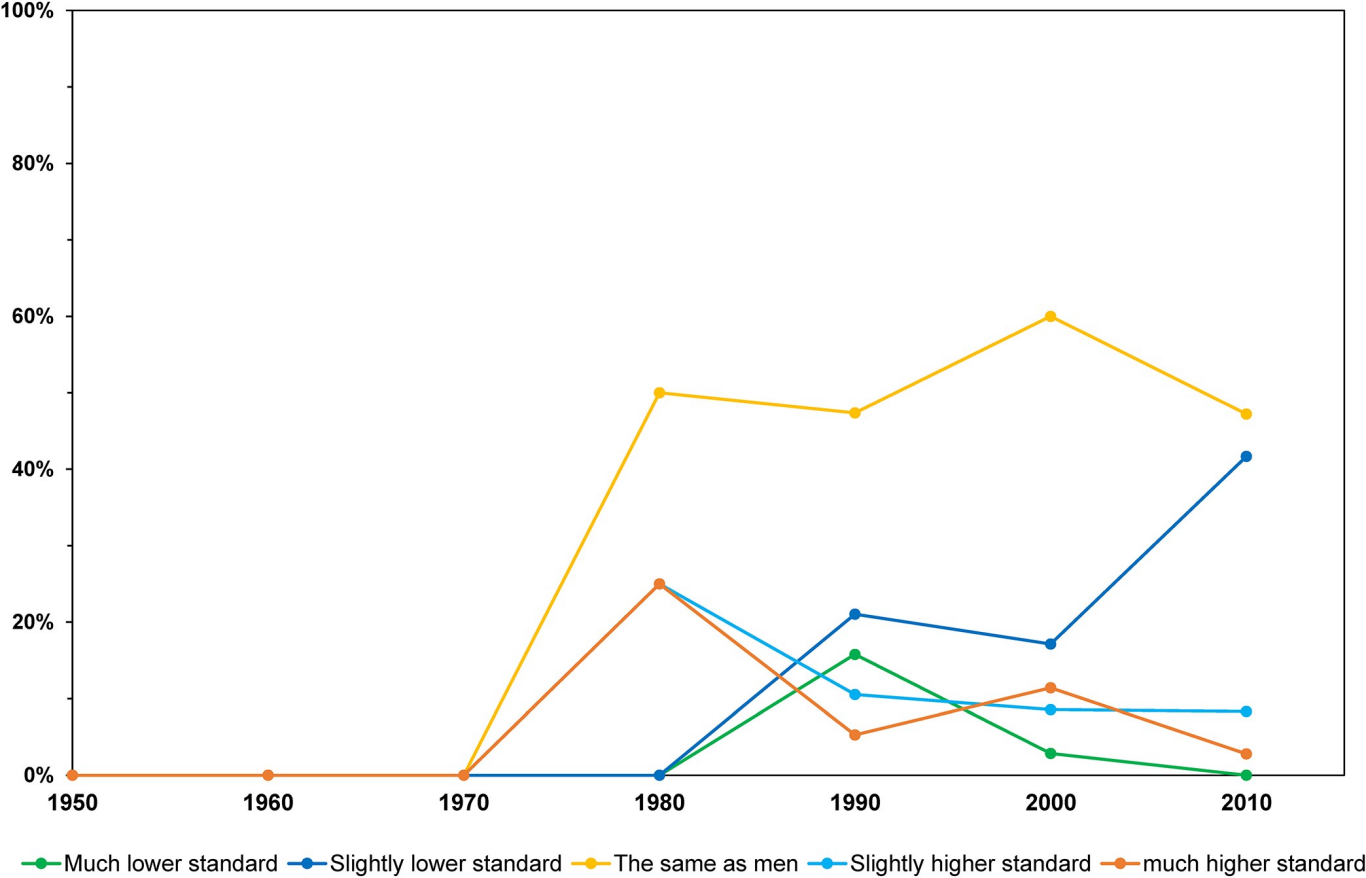
Perceptions of gender differentiation by decade in which fieldwork was undertaken.

Examining the data by decade reveals that 69% of women working in Antarctica in the 2010s reported no gender differentiation compared to 52% in the 1990s and 2000s. This difference perhaps points to policy and cultural shifts in the AAD as more women work in Antarctica over each decade (Fig 3).

The open-ended survey responses in which women discuss specific experiences are illuminating and more nuanced. Ten participants note that whilst duties are often shared, women are given menial tasks more often than men.

Certain male expeditioners expected me to do the cleanup in the field huts, cook meals, organize food stores….

In the field, gender differentiation was fine as I was the field leader. However, some of the male field workers were hesitant to cook, others were fine. As a general observation though, the male field workers were more resistant to cook than the females and some did have the expectation that the female field workers would cook for them. On the station, women were always allocated the domestic tasks, such as kitchen-hand during re-supply. Very rarely were they given tasks on the wharf to assist with resupply. This changed slightly when women were wintering expeditioners, rather than summering expeditioners.

These insights accord with research showing that gendered divisions of labor are experienced by women in the field [42]. Our data suggest that different physical work is required for men and women (e.g. women do the cooking) in line with cultural prescriptions of femininity. However, as noted below, women were also perceived to do more organizational work than men.

Socially women end up doing the bulk of the organization. I also saw a lot more organizational responsibility being undertaken by women generally. In the field I think that women often default to men, and in all other areas men default to letting women organize it. It's a system that's mutually beneficial, but often leaves women with a lot of work/responsibility…

Four participants responded to the question of barriers in Antarctic fieldwork with generalized experiences of cultural sexism and gender bias in the field.

…there are mental barriers–women are seen as less, and need to earn respect, rather than having it given as a common assumption like it seems to be when men turn up for the same job.

I believe as a woman you always have to prove yourself.

I found it part of the Antarctic culture to show that you could “cut it” by still working well with blatant sexism and not “cracking under pressure”.

Women [are] not seen as researchers (but) as women.

In these extracts, women describe difficulties inherent in working in male-dominated field environments. Given this masculinist culture, participants describe their experiences through the lens of cultural sexism [43]. According to Savigny, cultural sexism is a term that “combines the notion that sexism is an everyday, ordinary, occurrence, which takes place within masculinized hegemonic structures which interact with and create cultural norms and values…” [43]. In these contexts, women are undermined and are expected to not to “crack under pressure”. Open-ended survey responses highlight both the lack of women in leadership positions generally and the lack of support for existing women field leaders.

The women leaders appeared to be openly judged by their male counterparts.

I was a Field Leader and the weather was severe. I tried to get to a hut but it was too dangerous, another Field Leader came to me and said he was taking them anyway, I explained the conditions and that he would put the group in danger. He said he was fine and took them anyway. Later two people said that they were totally frightened and that they almost got bogged in a melt pool. So I felt ego came before safety to make me look incompetent.

… All the males who had field experience were automatically made trip leaders by our SL (Station Leader)…

…Less acceptance of the value of the project when female scientists are leading the field program.

When women and minority groups are persistently excluded in research/field cultures, masculinized leadership identities are reproduced [44]. This insight translates into an association of men/masculinity with competence in Australian Antarctic fieldwork, the effect of which is that women are rendered less intelligible to decision makers as potential leaders.

**Fig 4:**
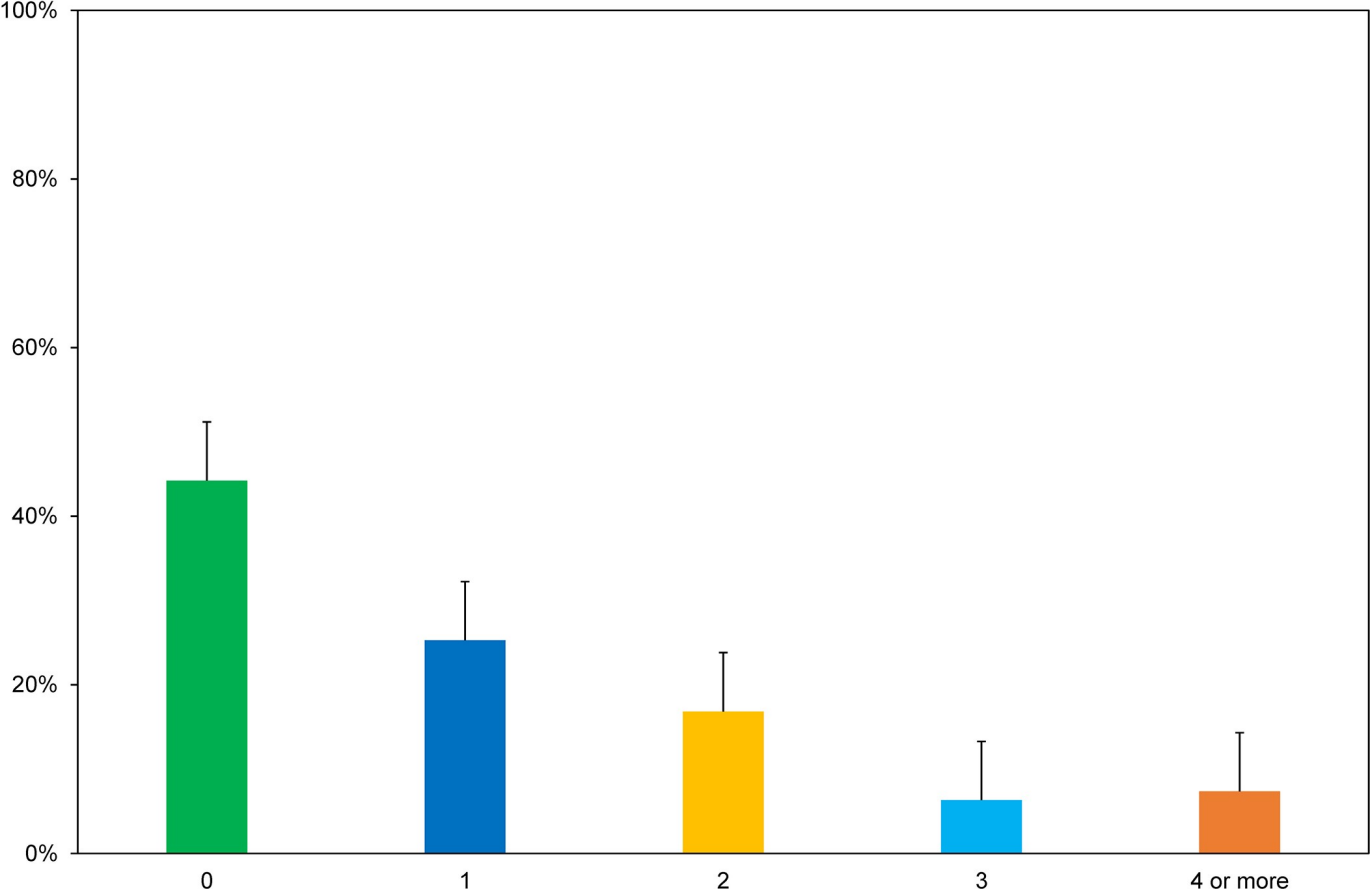
Number of participant field experiences in which science or field team leader was a woman.

Homosociality is often unacknowledged but can impair women’s ability to do fieldwork and lead [45]. Gender bias occurs in (white) masculinist/homophilic cultures where men advance their careers and gain power based on shared interests, sponsorship, and informal networks with other (white) men. Homophily is also problematic because it contributes to the homogeneity of organizations. For example, 44% of study participants (n = 42) had never worked with a female station or field team leader (Fig 4). As in the extracts above, leadership positions are filled by “cloning”, in which men appoint in their own image [46]. Importantly, as Holgersson [45] observes, “other social power relations such as class, ethnicity, race and sexuality also condition homosociality”.

#### Lack of opportunities and recognition

Science is an increasingly competitive field–there are few tenured jobs available and government funding of research is declining in much of the world. In Australia, job prospects for science graduates are the poorest despite ongoing calls for students to undertake STEMM degrees [47]. Given this context, a lack of adequate funding and opportunities to conduct research and/or do fieldwork in Antarctica remain key barriers for women in this study.

It’s becoming increasingly more difficult to get funding for Antarctic research…it is extremely difficult to get the fieldwork required and in trying to get other jobs, I felt that I am now pigeon holed into being an Antarctic researcher and not seen to be able to do anything else which has affected my career progression and ability to get postdocs.

Difficult to get fieldwork that pays decent money.

As demonstrated in the literature, women face specific challenges such as unconscious biases in this increasingly competitive context. For instance, women are often not included in informal networks in which information about promotion possibilities and job openings are exchanged [48]. Women tend to submit fewer grant applications and are also funded significantly less often in the UK, US, and Australia [49], while men tend to be favored in funding applications in relation to quality of the researcher and track records [50].

Decadal samples of Australian Antarctic Science Grants (Table 2), show that the percentage of grants with a woman as lead Chief Investigator (CI) is increasing but remains low at just 19% in recent years. Moreover, as the 2016 Antarctic Women Wikibomb event highlighted, successful women are recognized for their achievements less often than men [51]. For instance, women scientists comprise only 11% of the Scientific Committee on Antarctic Research (SCAR) medal winners [12] and 10% of Australian Antarctic Medal winners [52]. To be considered for the Australian Antarctic Medal, nominees must have spent an accumulated 12 months+ in Antarctica, which presents a structural disadvantage to women with caring responsibilities.

**Table 2:** Samples of one season per decade of Australian Antarctic Science Grants. In 1986–87, the 5% reflects that one woman had 3 projects [53]. CI refers to Chief Investigator.

Year of active project	1986–87	1996–97	2006–7	2016–17
% women as lead CI	5	12	14	19

#### Unwanted male attention/Sexual harassment

Survey participants provided positive feedback about the overall social environment of their most recent field experiences. Most respondents described the social environment of stations and ships with words including “enjoyable”, “comfortable”, “friendly”, and “life-changing”. However, in their open-ended responses to questions about gendered barriers, women identified that a primary challenge of field environments is unwanted attention from men due to the isolated, highly (hetero)sexualized environment. As Burns [54] observes, women are seen as “sexual hand-grenades”. As participants in this study attest, women are watched and scrutinized as potential sexual partners.

The Aurora Australis has been nicknamed the “Love Boat”…There is an expectation from some males that shenanigans (sexual relations) will be entered into.

With the small ratio of women to men it was tiring having to put up with all the men circling. It doesn't help that over summer all the animals are breeding and some of the humans just seem to follow suit.

Being in the minority, I was the only female on a return voyage full of randy males.

People felt the need to warn me that it (working on a station) could be difficult because you receive unwanted attention from men…Most winterers (fieldworkers who stay in Antarctica over winter) are men–after a winter of only or mostly male company, I was warned some of the men can become very interested in the company of the predominantly young female scientists coming in.

Here, heterosexuality is culturally hegemonic, and stories of the highly sexualized fieldwork environment are passed on to women before they even arrive in Antarctica [54]. Participant comments suggest that inappropriate or unwanted sexual behavior is a given in the field, and women must learn to cope with it [55]. The historical absence of women in the field (especially during winter) is positioned as a hardship for men; and the arrival of women into the isolated, confined environment is the catalyst for men to lose their self-control. As Flood [56] suggests, this problematic “patriarchal heterosexuality” cements the bonds between men and structures their relationships with women.

Sexual harassment is notable in experiences of women in polar science [57] and in STEMM fields broadly. Several high profile sexual harassment cases were revealed at the time that our survey was disseminated [24]. Seventy percent of survey participants reported being aware of the AAD’s Service Code of Personal Behavior [58], which explicitly prohibits any harassing behavior. Thirty-six percent of participants noted that they sometimes have observed colleagues making inappropriate or sexual remarks in the field. Sixty-three percent of participants had been on the receiving end of such remarks and most of these instances occurred when women were postgraduate students (40%) or technicians/field assistants (20%). One experienced participant remarked:

Having done (many) voyages to Antarctica in various roles…I have seen the general expeditioner population evolve due to changes in the selection criteria, especially for the tradesmen employed by the AAD. However, the selection of science projects does not always involve character selection criteria for the scientists involved. This makes the field research environment more volatile in terms of how women may be treated…I have also seen the workplace reporting processes improve, with the allocation of roles for workplace harassment officers, etc., so things are slowly improving it seems…From hear say, it seems harassment situations still occurring in a somewhat more insidious form.

Although participants who experienced harassment in the field generally knew how to report incidents (e.g. speak to the station leader), most (70%) remained silent. Below, five participants explain the complexities of reporting inappropriate behavior:

While I did not hear sexual comments, I was grabbed on the behind by one of the crew during a voyage. I was so shocked when it happened that all I could shout was "Hey!" while he snuck off. I mentioned it to some colleagues and they (female) had other experiences with the same person. But none of us mentioned it further. The hassle of reporting it and not being believed, being dragged through disciplinary meetings (if it ever got that far) and reading about other researchers experiences in reporting behavior far worse, made the prospect of reporting it not seem worthwhile for a grabbed behind.

I guess proactively make women aware that there is support for them if they are having problems with unwarranted attention as there was a perception when I was there that these situations were not dealt with openly i.e. women didn't feel they could speak up as they would have to deal with fall out especially if stuck on station with the person / people in question for another few months.

I shared a cabin back with a woman who had a horrific winter. She had been emotionally and possibly sexually abused. There was absolutely nothing done. I don't know if she reported it because there was no system for reporting that I knew of…

I did not think to report it at the time. When you're working 16 hours a day in a remote location—the general theme is to move on and get on with the work rather than make more work for yourself/others.

Most of what I have experienced is difficult to prove and becomes a "he said, she said"

Of those that experienced harassment, 49.5% of survey respondents took no action. The pragmatic approach of not reporting harassment implies that to advance, individual women need to adapt to the existing sexist culture or else they may suffer repercussions. This aligns with recent US research [59] revealing that women in STEMM often do not report harassment or sexism because it allows them to “blend in”; there is pressure on women to deny gender lest they risk their jobs.

As two participants noted below, reporting is particularly difficult due to the procedure and investigative channels:

I believe the AAD is under-prepared for problems arising in scientific research teams, with no impartial advice available on station. Students are reluctant to approach senior management directly with issues, as they feel it may worsen the situation, bring on unintended consequences and damage their future careers.

I found that it was easier and less confronting to report the harassment to a woman in a higher position of power than if it had been a man that I had had to report too.

Although reporting harassment can be challenging in any context, as in the extracts above, this is particularly acute in small field teams or in remote sites where it may not be possible for women to leave. Consequently, women may have to continue to live/work with their harassers for weeks while a complaint is investigated. The women in our study made it apparent in their open-ended comments that the onus is on women to make a complaint and that there is unacknowledged emotional labor associated with having to determine if a complaint is justified and worth the risk of “damaging their future careers”.

## Discussion

In this article, we provide a critical overview of exploratory survey data examining the research and remote fieldwork experiences of women working in the Australian Antarctic Program in recent decades. This study builds depth and breadth in the existing literature on scientific fieldwork. As we have argued, Antarctica is a unique context for studying fieldwork for women; although they face many of the recognized gendered barriers in STEMM identified in the literature, these barriers are compounded by challenges inherent in a remote polar fieldwork context. The lack of attention paid to issues of gender and sexuality in polar fieldwork specifically contributes to the invisibility and exclusion of women and other marginalized identities more broadly [60].

We highlighted five key themes that shape women’s experiences of Antarctic research and fieldwork including physical barriers, caring responsibilities/unpaid work, cultural sexism/gender bias, lack of opportunities/recognition, and unwanted male attention/sexual harassment. Often women’s default in this study was to de-legitimize research and fieldwork experiences by denying gender (e.g. we face no barriers, treated same as men)–but this belies their discursive descriptions about fieldwork. For instance, women’s experiences of physical barriers (e.g. having appropriate sized clothing, personal hygiene) have rarely been discussed in the existing literature but highlight the ways in which the ideal fieldwork participant is still discursively constructed as male and able-bodied. In this way, the image of the masculine Heroic Era figure prevails.

An important finding is that women in our sample, as in other STEMM fields, are doing a disproportionate amount of caring and unpaid employment-related work. Extended time away in remote locations over several seasons continues to be critical to a successful polar science career. Thus, the ideal scientific fieldworker is still represented as an unencumbered male whose private life does not impinge on his availability and commitment to fieldwork. Although women engage novel solutions to address the issue of extended time away (e.g. outsourcing labor to students/assistants), women can only do this with funding or their organizational context provides them with the resources to do so. Women on casual or fixed-term contracts do not have this option and may have to shift their career direction.

We argue that the image of fieldwork needs to change broadly–promoting local/smaller scale or shorter fieldwork expeditions are obvious solutions for researchers who cannot travel due to caring responsibilities [23]. However, we acknowledge that there are potential substantial costs involved in such changes. For instance, flying scientists to Antarctica for short periods is considerably more expensive. Thus, there are important financial implications for organizations to make fieldwork more accessible. However, we believe this is critical for all scientists to maintain active research agendas. Similarly, it is necessary to shift away from the attitude in certain scientific disciplines that rely on fieldwork that new knowledge requires new samples. Better sharing of resources, databases, and samples is important to organizations like SCAR and the Antarctic Treaty System as it reduces research impacts on Antarctica and adds value to existing collections. Attitudinal changes are important because the logistics of fieldwork can create inequality for women, undermining potential so advancement is less likely. As with caring responsibilities, public outreach also appears to be a highly gendered/feminized activity, which means that is de-legitimized if it is broadly associated with women scientists [41].

Women reported generalized experiences of sexism and gender bias in the field, many of which are commonly reported in STEMM. Participants note that they start fieldwork from a position of less credibility and women continually need to prove their competence to be accepted by men. Gender bias against women matters because the effects of bias and sexism accumulate over a woman’s career [43]. The cumulative effect of these practices is referred to as the glass ceiling, which comprises the deeply embedded, routine organizational policies and practices that hinder women’s career advancement [61]. These practices are concealed by their routine operation in “patterns of interaction, informal norms, networking, training, mentoring and evaluation” [61]. Although STEMM organizations may now be formally committed to gender equality, homophilic practices based on a masculinist fieldwork culture (e.g. all team leaders are men) can undercut these processes. Moreover, women in polar science are not only competing for credibility, they are also competing for resources and recognition. Women experience barriers in accessing Antarctic research funding, fieldwork opportunities, and recognition (e.g. prestigious national/international awards).

Although attitudes towards acceptable language and practices have shifted, sexual discrimination, harassment, and unwanted sexual attention remain issues of concern. More than half of participants report experiencing inappropriate behavior during fieldwork. Because the AAD’s code of conduct is not regularly enforced in the field, most women did not report harassment given the bureaucratic difficulties and potential stigma associated with doing so. Reporting can be challenging in small field teams or remote sites due to lack of access to appropriate people to make reports or it may be unclear who to report harassment to. Furthermore, it also may not be possible to leave the site. Thus, women may have to live/work with their harassers for weeks until any action is taken.

As demonstrated in other STEMM fields, women often cope with these experiences silently. This is a problem because silence reinforces problematic heterosexual masculine norms [55]. Ensuring a diversity of people to whom women can report inappropriate behavior and simplifying reporting procedures can make a difference. For instance, promoting, educating and supporting “champions of change” within the AAD who are clearly identified to all expeditioners during induction/training can be a critical step forward [62]. Sexual harassment emerges from power imbalances. Thus, much of the harassment in our sample happened when women were in roles with less organizational power (e.g. postgraduate student). Accounting for how identity (e.g. gender, race, sexuality) structures power relationships is essential in highlighting sources of power inequality between women and men in the field and should be openly discussed prior to any expedition and used to build a supportive culture.

Additionally, sexual harassment tends to be seen through an exclusively heterosexual lens. Bringing the experiences of gender and sexuality diverse people into focus raises significant questions about polar fieldwork. Scientists are stereotypically represented as straight white men, and research suggests that there are “rigid expectations of gender and sexuality” in many STEMM workplaces [60]. Heterosexism, or the normalization of heterosexuality in the workplace, can significantly affect whether gender and sexuality diverse people are “out” in the workplace [63]. Not being “out” can compromise an employee’s wellbeing, career satisfaction, and productivity [63]. The recent launch of Pride in Polar Research, a joint initiative of SCAR and International Arctic Science Committee (IASC) researchers focused on drawing together the LGBTQ+ community and Allies (friends and supporters), is a critical step forward in supporting and making visible the spectrum of identities within the polar science community.

It is crucial that future research uses an intersectional approach and specifically engages with gender and sexuality diverse polar fieldworkers to ensure that their experiences are addressed in local, national, and international equity initiatives. A limitation of the current study is that the experiences of gender and sexuality diverse people in polar field environments are not represented. It is important to address “heterosexist harassment”, defined as “insensitive verbal or symbolic behaviors that convey animosity towards non-heterosexuality” that can include verbal, physical, and sexual assault/harassment [64]. In our survey, very few women identified as gender and/or sexuality diverse and none shared specific experiences in open-response questions. Given the cisgender, heteronormative character of Australian Antarctic science broadly and of our sample specifically, it is unsurprising that participants may have been hesitant to volunteer information about being gender and/or sexuality diverse if they are not “out” at work or fear being too easily identified.

### Moving forward in polar research and beyond

Currently, Australian policies and programs aimed at improving gender diversity in STEMM are based on making the workplace culture more “woman-friendly” by introducing gender-neutral hiring, sensitivity training, parental leave policies and flexible work arrangements [65]. Whilst important, these strategies may simply reinstate continuing inequalities as “women’s problems”. Similar strategies are being directed towards closing the leadership gap. To address the leaks in the gender pipeline, efforts to grow the leadership pool and diversify leadership appointments has led to many leadership programs targeting women. The growth in women’s leadership programs is a function of an assumption that men’s dominance in positions of leadership is natural whereas women require specialized programs to develop the necessary skills to be recognized for promotion. Structural inequality is not a problem that individual women should be expected to fix, either in Australian Antarctic research or in STEMM broadly.

It is important to note that although Australian Antarctic science must be undertaken through the AAD, responses to gendered fieldwork issues may differ between the AAD and the universities or other institutions where women also work. For example, The Athena SWAN (AS) Charter for Women in Science is a gender equity award scheme that began in the United Kingdom in 2005 and expanded to Australia in 2014 [66]. Gendered barriers in polar research and fieldwork vary cross-culturally. However, the type of institutional data gathered as part of AS is not readily compiled across the polar regions nor is there a coherent process for action planning. Polar organizations like the Council of Managers of National Antarctic Programs (COMNAP) might consider the implementation of a Polar AS process to ensure that fieldwork issues are being discussed and managed at the highest levels and that there are incentives for agencies to change their organizational practices.

For polar organizations, commitment to addressing inequalities is a critical first step but substantive change requires ongoing discussion with diverse group of women about their research and fieldwork experiences, financial investment, and long-term commitment. This study highlights the value of institutional and social policies that promote supportive working environments for women and other marginalized groups as critical moves in enhancing productivity and inclusiveness. Future research might draw on these initial findings and adapt the survey to improve the AAD’s current organizational policies and to build a culture of change. Furthermore, we encourage research that extends to other nations’ Antarctic research communities and to survey polar fieldworkers of all genders. We also can see value in creating a survey for women working in remote field sites in locations such as the Arctic to provide comparative data. Moreover, we suggest findings could be strengthened with in-depth qualitative interviews and/or focus groups with women and men working in and managing polar research about their experiences. The image of the hero striding out into the wilderness needs to be relegated to the past.

## Supporting information

S1 File Antarctic women survey. (PDF)
