# Supplementary material for: “Antarctica just has this hero factor…”: Gendered barriers to Australian Antarctic research and remote fieldwork
Source: PLoS One. 2019 Jan 16;14(1):e0209983. doi: 10.1371/journal.pone.0209983 (PMC6334902; doi:10.1371/journal.pone.0209983)
Supplement: S1 File — (PDF) [file pone.0209983.s001.pdf]

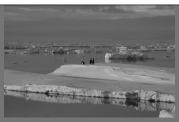

WOMEN IN AUSTRALIAN ANTARCTIC RESEARCH SURVEY

OCEANIC CULTURES AND CONNECTIONS PROJECT: GENDER EQUITY IN ANTARCTIC RESEARCH

Thank you for your interest in a current research project about women in Australian Antarctic research. This study is being conducted by Dr. Meredith Nash, A/Prof Mary-Anne Lea, Dr. Justine Shaw, Professor Matt King, Dr. Narissa Bax, and Hanne Nielsen. The University of Tasmania's Human Ethics Committee has approved this project. This study is being funded by a grant from the University of Tasmania.

What is the purpose of this study?
The proposed investigation will provide important data about women working in Australian Antarctic science. Your participation in this survey will provide information on how the government and relevant polar organisations can better support women. The results of this research will potentially stimulate a broader conversation about mentoring, fieldwork, and support of women in Australian Antarctic science.
This project has two aims:
1. To describe the social characteristics of women working in Australian Antarctic research.
2. To explore the organisational and fieldwork experiences of women working in Australian Antarctic research.

Why have I been invited to participate in this study?
You are eligible to participate in this study because you are a woman working now or in the past in Australian Antarctic research. You are aged 18 or above. You have been to Antarctica at least once with the Australian Antarctic science program (from the 1950s to present), either on a research vessel to the Southern Ocean, to a research base or field camp, including on Macquarie and Heard islands.

What does this study involve?
Your participation in this project will involve completing an online questionnaire in your own time. In order to determine your background and your experiences of Australian Antarctic research, questions are presented in a straightforward manner, requiring you to select from closed-choice responses. You will also be able to respond to open text questions. It will take approximately 10-15 minutes to complete the full survey. All information provided by you will remain anonymous.
Your involvement in this study is voluntary. While we would be pleased to have you participate, we respect your right to decline. There will be no consequences to you if you decide not to participate. If you decide to discontinue participation in this project at any time, you may do so by contacting the researchers, and will not be required to provide an explanation. All of the research data will be kept in password protected computer files and will be destroyed five years from the first date of publication.

Do I have to participate?
Your involvement in this study is voluntary. While we would be pleased to have you participate, we respect your right to decline. There will be no consequences to you if you decide not to

participate. If you decide to discontinue participation whilst completing the survey, you may close your Internet browser – none of your responses will be recorded. If you wish to withdraw your data after completing the survey, you may do so by contacting the researchers. You will not be required to provide an explanation.

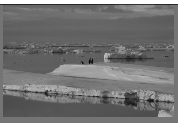

OCEANIC CULTURES AND CONNECTIONS PROJECT: GENDER EQUITY IN ANTARCTIC RESEARCH

This study has been approved by the Tasmanian Social Science Human Research Ethics Committee. If you have concerns or complaints about the conduct of this study you should contact the Executive Officer of the HREC (Tasmania) Network on (03) 6226 7479 or email human.ethics@utas.edu.au.

This research is low risk but you may experience some discomfort. For instance, you will be asked about sexual harassment experiences you may have had in the past at work, albeit in a general way. These questions may be triggering, and stimulation of these memories may make you uncomfortable. If you are at all worried that thinking about or sharing your experiences about sexual harassment would be too mentally harmful, please do not continue with this survey.

Should you require further support, you may contact Lifeline on their toll free number: (+61) 13 11 14 (24 hours). Further resources are available on their website and you can contact them online at www.lifeline.org.au/get-help/online-services/crisis-chat (7pm - 4am, 7 days a week).

If you would like to discuss any aspect of this study please feel free to contact Dr. Meredith Nash on (03) 6226 2715 or Meredith.Nash@utas.edu.au or Dr. Narissa Bax on baxn@utas.edu.au.

Thank you very much for taking the time to read this information. We hope you decide to participate in this important research and share the link with others in your network. If you wish to participate, please click 'yes' below, which will direct you to the survey.

Terms and Conditions

1. I have read and understand the Project Information for this project.
2. The nature and possible effects of the study have been explained to me.
3. I understand that the study involves completing an online questionnaire. I understand that all data collected will focus on women’s experiences of Australian Antarctic research.
4. I understand that all research data will be securely stored on the University of Tasmania premises for five years from the first publication, and then will be securely destroyed.
5. Any questions that I have asked have been answered to my satisfaction.

6. I agree that research data gathered from me for the study (i.e. data obtained from the questionnaire) may be published provided that I cannot be individually identified as a participant.
7. I understand that I will be given the option to identify myself on the survey and that any information I supply to the researcher will be used only for the purposes of the research.
8. I agree to participate in this investigation and understand that I may withdraw at any time without any effect, and if so wish, may request that any data I have supplied to date be withdrawn from the research.

* 1. I have read the Terms and Conditions above and consent to participate in this project by completing this questionnaire.

◯ Yes

◯ No

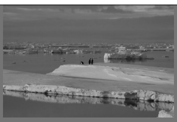

2. Are you currently working in Antarctic science?

- ○ Yes
- ○ No

3. In what decade did you begin working in Antarctic Science?

- ○ 1950s
- ○ 1960s
- ○ 1970s
- ○ 1980s
- ○ 1990s
- ○ 2000s
- ○ 2010s

4. What year did you last work in Antarctica?

5. Do you undertake fieldwork in your research?

- ○ Yes
- ○ No
- ○ I did in the past, but not anymore

6. What are the barriers (if any) to doing Antarctic fieldwork for women?

7. If you no longer do fieldwork, please explain why this is the case and how it has affected your career/research?

8. How many different stations, field camps, or marine science voyages have you participated in?

- ○ 1
- ○ 2
- ○ 3
- ○ 4 or more

9. At how many locations has the science leader or field team leader been a woman?

- ◯ 0
- ◯ 1
- ◯ 2
- ◯ 3
- ◯ 4 or more

10. Think back to your most recent field experience. What was the female:male ratio of researchers at the field site:

11. Whilst working within an Antarctic field team how valued was your input regarding the approach, methodology and implementation in your most recent field experience?

- ◯ not at all valued
- ◯ occasionally valued
- ◯ moderately valued
- ◯ very valued
- ◯ highly valued

12. To what standard do you think you were you held compared to male team participants in your most recent field experience (e.g. in terms of research capability, competence in the outdoors, responsibilities, etc)?

- ◯ much lower standard
- ◯ slightly lower standard
- ◯ the same as men
- ◯ slightly higher standard
- ◯ much higher standard

13. How gender differentiated was your most recent field experience? Examples would be that men and women do separate activities with their leisure time, or that bureaucratic or cleaning tasks get allocated disproportionately between genders.

- ◯ not differentiated
- ◯ slightly differentiated
- ◯ moderately differentiated
- ◯ very differentiated
- ◯ completely differentiated

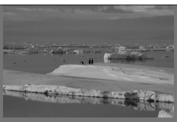

14. If you observed gender differentiation, please describe what sort of differentiation was observed.

15. If it is possible to do so, how might field camp/station/expeditioner code of conduct protocols and guidelines be improved to address gender bias?

16. How would you describe the social environment during your most recent field experience (please specify the location and if this was on station, ship, at field sites)?

17. Would you describe yourself as a leader?

- Yes
- No

18. What are the qualities of a good leader?

19. Have you ever received an award for being a woman in science or been promoted as part of an equity initiative?

- Yes
- No

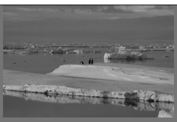

20. If yes, was it ever implied either implicitly or explicitly that you were only given that job/award because you are a woman?

- ◯ Yes
- ◯ No

21. Please provide more details if you wish.

[ ]

22. Do you view the grants and schemes in Science, Technology, Engineering, Mathematics, and Medicine (STEMM) available for women as positive or negative?

- ◯ Positive
- ◯ Negative
- ◯ Other

23. Has gender affected your ability to apply for or receive funding?

- ◯ Gender has affected my ability to apply for and receive funding.
- ◯ Gender has affected only my ability to apply for funding.
- ◯ Gender has affected only my ability to receive funding.
- ◯ Gender has not affected my ability to apply for or receive funding.

24. Has your work ever been devalued because of your gender?

- ◯ Yes
- ◯ No

25. If yes, what was the context (e.g., shorter, less praise-worthy letters of recommendation; fewer research grants, awards, and invitations to speak at conferences; and lower citation rates etc), and has this devaluation had repercussions in terms of your career goals?

26. Do you currently have access to a mentor, or mentoring program within your organisation?

- Yes
- No

27. If you have spent time with a mentor, how has the mentorship contributed to your career in Australian Antarctic Science?

28. In an Antarctic Science context, what are your main support networks? Do you feel sufficiently supported in your work environment? If not, how could the level of support be improved?

29. Please briefly describe any caring responsibilities you have. This may include for children, adults, or animals.

30. Have your caring responsibilities prevented you from going South?

- Yes
- No

31. How many hours do you dedicate to caring responsibilities per week (e.g. children, elderly parents, animals)?

- Less than 5 hours
- 1 - 5 hours
- 5 - 10 hours
- 10 - 15 hours
- 15 - 20 hours
- 20+ hours

32. How many hours do you dedicate to your career per week that are unpaid?

- ◯ Less than 5 hours
- ◯ 1 - 5 hours
- ◯ 5 - 10 hours
- ◯ 10 - 15 hours
- ◯ 15 - 20 hours
- ◯ 20+ hours

33. Describe this unpaid work (e.g., grant writing, public outreach, volunteer work experience and promotional opportunities etc).

34. Why do you undertake unpaid work?

35. Are you aware of, and informed about the content of the code of conduct outlined by the Australian Antarctic Program?

- ◯ Yes
- ◯ No
- ◯ I don't know

36. Did any of the field sites at which you have worked or been a student have a sexual harassment policy?

- ◯ Yes
- ◯ No
- ◯ I don't know

37. How often did you observe other field researchers and colleagues making inappropriate or sexual remarks?

- ◯ Always
- ◯ Often
- ◯ Sometimes
- ◯ Rarely
- ◯ Never

38. Have you ever personally experienced inappropriate or sexual remarks, comments about physical beauty, cognitive sex differences, or other jokes, whilst down south? (If you have had more than one experience, the most notable to you.)

- ◯ Yes
- ◯ No
- ◯ I don't know

39. If you answered yes, what was your position at the time of the incident?

- ◯ Undergraduate student
- ◯ Postgraduate student
- ◯ Technician or field assistant
- ◯ Postdoctoral fellow
- ◯ Tenured academic
- ◯ Employed field scientist
- ◯ Other

40. If you answered yes to question 37 was there a mechanism for you to easily report this harassment?

- ◯ Yes
- ◯ No
- ◯ I don't know

41. If so, what was the mechanism?

[ ]

42. If so, did you report it?

- ◯ Yes
- ◯ No
- ◯ I don't know

43. If you reported it, how satisfied were you with the outcome?

- ◯ very dissatisfied
- ◯ dissatisfied
- ◯ neutral
- ◯ satisfied

44. What is your ethnicity? (i.e. your feeling of belonging and attachment to a distinct group of larger population that shares your ancestry, skin colour, language or religion). For example, South-east Asian, African, Latino/Hispanic etc.

[ ]

45. Country of origin

[ ]

46. What is your age?

[ ]

47. What is your highest attained educational level

- ◯ High school
- ◯ High School/ currently completing University Bachelors Degree Bachelors Degree without Honours
- ◯ Bachelor's Degree with Honours
- ◯ Graduate Certificate
- ◯ Masters by coursework
- ◯ Masters by research
- ◯ Doctorate by coursework and research
- ◯ Doctorate of Philosophy

48. Current status

- ◯ Undergraduate student
- ◯ Postgraduate student
- ◯ Technician or field assistant
- ◯ Other (please specify)
- ◯ Postdoctoral fellow
- ◯ Government Scientist
- ◯ Professor

[ ]

49. Please indicate the sector of your main employment

- Private
- Government
- University
- Other (please specify)

50. Your subfield (if applicable, all that apply):

- Atmospheric Science
- Biology
- Environmental Science
- Geology
- Glaciology
- History
- Marine Science
- Meteorology
- National Parks
- Oceanography
- Social science
- Zoology
- Other (please specify)

51. What is your annual salary (including bonuses and commissions) in Australian dollars?

- $0 - $25,000
- $25,001 - $50,000
- $50,001 - $75,000
- $75,001 - $100,000
- $100,001 - $125,000
- $125,001 - $150,000
- $150,001 - $175,000
- $175,001 - $200,000
- $200,001+
- I prefer not to answer

52. Which best describes your current relationship status?

- Married
- Living in a de facto marriage/relationship
- Separated
- Divorced
- Widowed
- Never married
- Single
- I prefer not to answer

53. How many children (younger than 18 years) are living in the house with you? (If you have no children, answer zero [0].

54. Do you identify as___________

- Lesbian
- Gay
- Bisexual
- Transgender
- Intersex
- Queer
- Heterosexual
- Other
- I prefer not to answer

55. Do you have any additional comments?

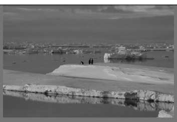

WOMEN IN AUSTRALIAN ANTARCTIC RESEARCH SURVEY

Thank you for participating in this survey!

Please provide your name and email address if you would like to be entered into a draw to win one of two $100 Coles/Myer vouchers. This information will not be linked to your survey responses.

56. Name

57. Email address
